# RETRACTED: Li et al. TRIM10 Is Downregulated in Acute Myeloid Leukemia and Plays a Tumor Suppressive Role via Regulating NF-κB Pathway. *Cancers* 2023, *15*, 417

**DOI:** 10.3390/cancers18101507

**Published:** 2026-05-08

**Authors:** Lin Li, Qi Li, Zhengrong Zou, Zoufang Huang, Yijian Chen

**Affiliations:** 1 Suzhou Medical College of Soochow University, Suzhou 215123, China; 18979740225@163.com; 2 Department of Hematology, The First Affiliated Hospital of Gannan Medical University, Ganzhou 341000, China; nfyyjsjj@126.com; 3 Basic Medicine Department, Chuxiong Medical and Pharmaceutical College, Chuxiong 675005, China; 15559705116@163.com; 4 Department of Emergency, The First Affiliated Hospital of Gannan Medical University, Ganzhou 341000, China; zzrboy87@163.com

The journal retracts the article “TRIM10 is downregulated in acute myeloid leukemia and plays a tumor suppressive role via regulating NF-κB pathway” [1] cited above.

Following publication, concerns were brought to the attention of the Editorial Office regarding the integrity of images presented in this publication [1].

Adhering to our standard procedure, an investigation was conducted by the Editorial Office and Editorial Board that identified indications of inappropriate editing and duplication between Figure 1C in [1] and Figure 3I,K of an earlier publication [2], produced by a different research group. While the authors collaborated within this process, raw material meeting the journal’s requirements for original images (https://www.mdpi.com/journal/pharmaceuticals/instructions#oriimages) could not be provided for Editorial Board Evaluation. Consequently, the Editorial Board has lost confidence in the reliability of the findings and has decided to retract this publication, as per MDPI’s retraction policy (https://www.mdpi.com/ethics#_bookmark30).

This retraction was approved by the Editor-in-Chief of the journal *Cancers*. The authors did not provide a comment on this decision.
